# MFH Mimic in Breast: A High-Grade Malignant Phyllodes Tumor

**DOI:** 10.1155/2012/835687

**Published:** 2012-10-17

**Authors:** A. L. Hemalatha, V. Sumana Sindhuram, U. Asha

**Affiliations:** Department of Pathology, Mysore Medical College & Research Institute, Irwin Road, Mysore 570001, India

## Abstract

Malignant phyllodes tumor is usually diagnosed by the presence of benign duct-like epithelium and malignant mesenchymal tissue. In addition to the usual fibrosarcomatous features, the mesenchymal component may show areas resembling osteogenic sarcoma, chondrosarcoma, liposarcoma, leiomyosarcoma, rhabdomyosarcoma, malignant mesenchymoma, and, very rarely, malignant fibrous histiocytoma. We present one such rare case of malignant phyllodes tumor with malignant fibrous histiocytoma-like stromal differentiation.

## 1. Introduction 

Phyllodes tumor of the breast is an uncommon biphasic fibroepithelial neoplasm that accounts for less than 1% of overall breast neoplasms [1]. In addition to stromal overgrowth and hypercellularity, malignant phyllodes tumors are characterized by infiltrative margins, atypical mitoses, necrosis, hemorrhage, and atypia. High-grade phyllodes tumor is a rare but aggressive breast malignancy and forms approximately 25% of all phyllodes tumors [2]. Malignant stromal transformation in phyllodes tumor is usually of the fibrosarcomatous type but sometimes the mesenchymal component may show areas resembling osteogenic sarcoma, chondrosarcoma, liposarcoma, leiomyosarcoma, and rhabdomyosarcoma. Very rarely, MFH-like differentiation may occur giving rise to a mistaken diagnosis of primary MFH of the breast which is extremely uncommon [3]. The present case deals with one such unusual MFH-like presentation of malignant phyllodes tumor in a 30-year-old woman.

## 2. Case Report

A 30-year-old multiparous female presented with a palpable, centrally situated, firm to hard mass in the upper outer quadrant of right breast of 2 years' duration. Multiple, firm, satellite nodules were palpable along the medial and inferior aspects of the lump. FNAC showed a highly cellular smear comprised of pleomorphic tumor cells with scant cytoplasm and bizarre nuclei in singles and tiny clusters. Abundant uni-, bi- and multinucleated osteoclast—like giant cells and atypical mitotic figures were seen in a background of monolayered sheets of benign ductal epithelial cells. A cytological diagnosis of malignant breast neoplasm was followed by right mastectomy with axillary exploration.

## 3. Pathological Findings

### 3.1. Gross Examination

**Figure 1:**
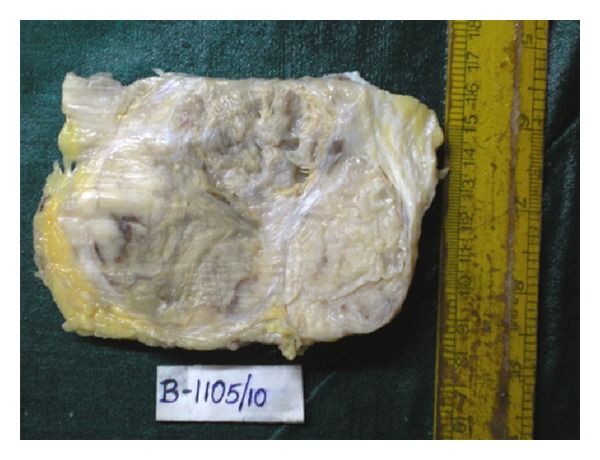
Cut section of specimen showing large grey-white tumor with central cystic area.

**Figure 2:**
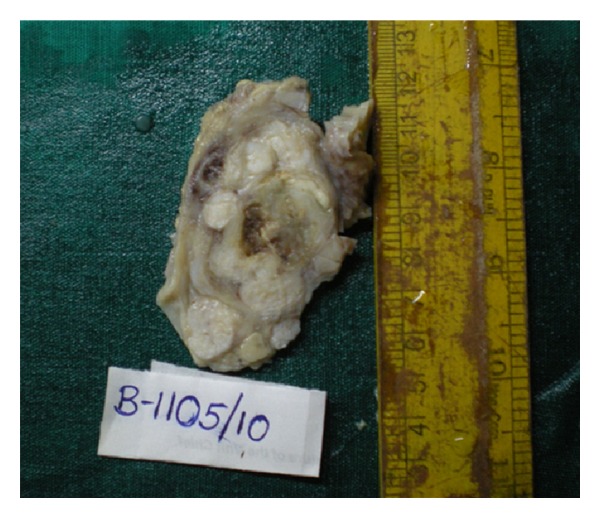
Cut section of specimen showing a large tumor with peripheral area of tumor showing satellite nodules.

Serial sectioning of the mastectomy specimen revealed a large, grey-white, lobulated mass involving the entire breast and extending up to the deep surgical margin (Figure 1). The central area of the mass showed cystic degeneration and the periphery of the mass showed multiple grey-white, solid, well-circumscribed nodules (Figure 2).

### 3.2. Microscopic Examination

**Figure 3:**
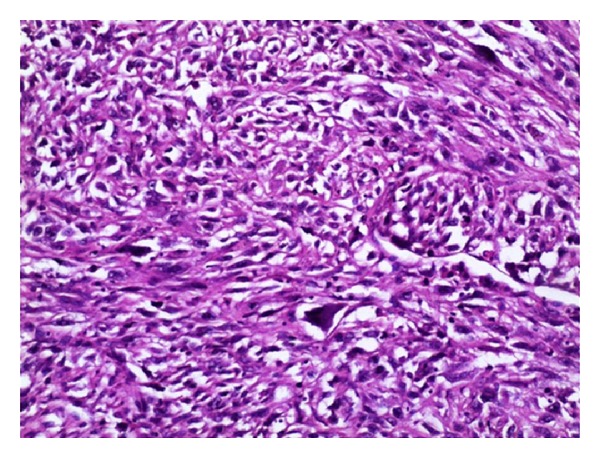
Highly pleomorphic spindly tumor cells with abundant atypical mitoses; (H&E 40x).

**Figure 4:**
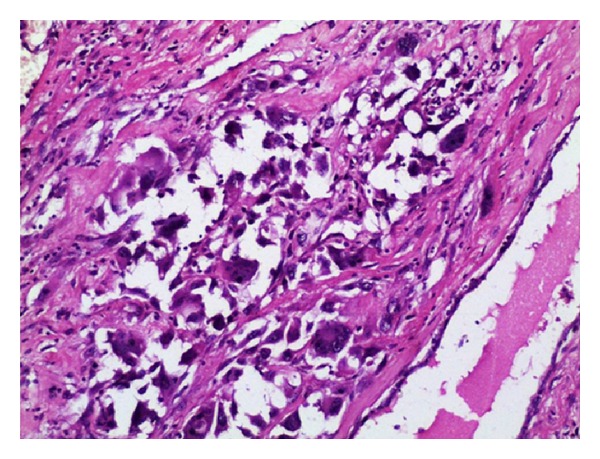
Malignant phyllodes tumor with multinucleated giant cells mimicking MFH; (H&E 40x).

A highly cellular tumor comprised of predominantly spindle cells with pleomorphic, hyperchromatic nuclei and plenty of bizarre mitoses (Figure 3). A few uni-, bi-, multinucleated osteoclast–like giant cells were also seen (Figure 4). Occasional benign ducts were identified. The peripheral nodular areas showed compressed leaf-like benign ducts enclosed by proliferating benign stromal fragments. A histopathological diagnosis of high grade malignant phyllodes tumor was offered.

**IHC**

Anaplastic spindle cells were negative for Cytokeratin (AE1/AE3) and positive for Vimentin.

## 4. Discussion

Most patients with phyllodes tumor are middle-aged or elderly and its occurrence in younger patients as in the present case is exceptional. Usually, malignant transformation with fibrosarcomatous type of differentiation is seen in about 30% of phyllodes tumors. Rarely, heterologous sarcomatous differentiation may be seen. The neoplastic stromal component may be monomorphic or highly pleomorphic with an appearance reminiscent of MFH, fibrosarcoma, or liposarcoma.

In the present case, primary MFH was the most advocated differential diagnosis due to the presence of highly neoplastic stromal component admixed with uni-, bi- and multinucleated osteoclast-like giant cells which mimicked MFH in focal areas. MFH was ruled out considering the benign phyllodes pattern in the satellite nodules and the absence of prototypic storiform pattern diagnostic of MFH. Multi-nucleated osteoclast-like giant cells associated with malignant phyllodes tumor are nonneoplastic in origin and are considered to be modified histiocytes probably in reaction to extracellular material produced by the tumor cells [4]. Heterologous elements such as metaplastic cartilage, bone, or exceptionally skeletal muscle may be present in malignant phyllodes tumor. None of these features were observed in the present case. Metaplastic carcinoma with spindle cell differentiation was ruled out considering the absence of malignant glandular elements and cytokeratin negativity in the tumor cells. Cytokeratin negativity and vimentin positivity confirmed the malignant mesenchymal nature of the tumor in the present case.

Metastasis in malignant phyllodes tumor is primarily hematogenous, with lung, pleura, and bone, being the common sites. It has been reported to occur at a rate of 13% in ten years [5]. As malignant phyllodes tumor usually spreads by a hematogenous rather than a lymphatic route, axillary lymph node dissection is generally not recommended. But in the present case, since the mastectomy followed a cytological diagnosis of malignant breast neoplasm where a subtyping of tumor was not possible, axillary lymph node resection was undertaken.

The poor prognostic factors in the present case include higher grade of tumor, large tumor size, and tumor necrosis. Early detection is associated with better prognosis. 

## 5. Conclusion

Malignant phyllodes tumor is usually associated with fibrosarcomatous type of stromal differentiation but very rarely MFH-like differentiation may occur giving rise to a diagnostic dilemma. A definite distinction is imperative considering the better prognostic implications of malignant phyllodes tumor and different management options available for the two entities.
